# Highly Efficient Elimination of Colorectal Tumor-Initiating Cells by an EpCAM/CD3-Bispecific Antibody Engaging Human T Cells

**DOI:** 10.1371/journal.pone.0013474

**Published:** 2010-10-18

**Authors:** Ines Herrmann, Patrick A. Baeuerle, Matthias Friedrich, Alexander Murr, Susanne Filusch, Dominik Rüttinger, Mariam W. Majdoub, Sherven Sharma, Peter Kufer, Tobias Raum, Markus Münz

**Affiliations:** 1 Micromet AG, Munich, Germany; 2 Micromet, Inc., Bethesda, Maryland, United States of America; 3 Celprogen Inc, San Pedro, California, United States of America; 4 Department of Medicine, University of California Los Angeles, Los Angeles, California, United States of America; 5 Molecular Gene Medicine Laboratory, Greater Los Angeles Healthcare System, Los Angeles, California, United States of America; Ludwig-Maximilians-Universität München, Germany

## Abstract

With their resistance to genotoxic and anti-proliferative drugs and potential to grow tumors and metastases from very few cells, cancer stem or tumor-initiating cells (TICs) are a severe limitation for the treatment of cancer by conventional therapies. Here, we explored whether human T cells that are redirected via an EpCAM/CD3-bispecific antibody called MT110 can lyse colorectal TICs and prevent tumor growth from TICs. MT110 recognizes EpCAM, a cell adhesion molecule expressed on TICs from diverse human carcinoma, which was recently shown to promote tumor growth through engagement of elements of the *wnt* pathway. MT110 was highly potent in mediating complete redirected lysis of KRAS-, PI3 kinase- and BRAF-mutated colorectal TICs, as demonstrated in a soft agar assay. In immunodeficient mice, MT110 prevented growth of tumors from a 5,000-fold excess of a minimally tumorigenic TIC dose. T cells engaged by MT110 may provide a potent therapeutic means to eradicate TICs and bulk tumor cells derived thereof.

## Introduction

While mortalities of heart disease, stroke and infectious diseases (flu and pneumonia) have declined over the past 55 years by 64, 74 and 58%, overall cancer mortality did only decline by 5% (National Center for Health Statistics). One reason may be the resistance of so called cancer stem or tumor-initiating cells (TICs) to standard cancer therapies. This highly tumorigenic subpopulation of cancer cells is difficult to detect and resistant to many chemotherapeutic approaches due to overexpression of detoxifying enzymes and multidrug resistance pumps, preference for hypoxic niches and low proliferation rate. The significance of TICs for cancer therapy and biology is therefore under intense research [1]–[4]. Should the concept of TICs hold up, novel therapies aiming at their elimination may treat cancer with improved outcome, if not with a curative effect.

TICs have now been identified and characterized in numerous human malignancies. Several laboratories have isolated TICs, e.g., from colorectal and pancreatic tumors, by using antibodies specific for epithelial cell adhesion molecule (EpCAM; also called ESA) [5], [6]. Furthermore, the expression of EpCAM and CD44 was shown to track with the tumorigenic phenotype of such cells [5], [7]–[10]. EpCAM is frequently expressed at high levels on primary tumors and metastases of most human adenocarcinoma [11], [12]. In several human malignancies, including breast, ovarian, ampullary pancreas, gall bladder and liver cancers, EpCAM overexpression correlates with a poor survival prognosis of patients [13]–[17]. EpCAM has recently been described as a cancer stem cell marker expressed together with CD44, CD133, and CD166 [18]–[20].

One reason why TICs and their progeny may express EpCAM is that the adhesion molecule can be activated by regulated intra-membrane proteolysis allowing it to function as signaling protein and proto-oncogene [21], [22]. The released intracellular domain of EpCAM, called EpICD, has been shown to form a nuclear complex composed of FHL-2, β-catenin and transcription factor TCF/LEF, which is involved in expression of c-myc and cyclin genes. Overexpression of full-length EpCAM or EpICD in quiescent cells elicits tumor formation. EpCAM has been selected as target for many antibody- and vaccine-based therapeutic approaches of which several are in clinical development [11], [23]. A trifunctional anti-EpCAM antibody has recently obtained market approval in Europe. Certain normal epithelial tissues and embryonic stem cells express EpCAM [24]–[26], but there is evidence to suggest that EpCAM on normal epithelial tissues is largely sequestered while it is accessible on the surface of cancer cells [27], [28].

MT110 is a T cell-engaging antibody construct of the BiTE class i.e. bispecific T cell engager with dual specificity for EpCAM and CD3 [29]. The principle of BiTE antibodies has been reviewed in detail [30]–[33]. BiTE antibodies enable formation of a cytolytic synapse between any cytotoxic T cell and a target cell binding the BiTE antibody. This will fully activate T cells for redirected lysis involving production of granzyme B, proliferation, and adoption of a serial lysis mode [34]–[36]. MT110 and related EpCAM-specific BiTE antibodies showed high anti-tumor activity in diverse animal models [29], [37]–[39]. A CD19/CD3-bispecific BiTE antibody called blinatumomab showed high anti-tumor activity in relapsed non-Hodgkin's lymphoma patients [40], providing clinical proof of concept for the therapeutic principle of BiTE antibodies. MT110 is currently tested in a dose-escalating phase 1 clinical trial in patients with lung or gastrointestinal cancers for safety and initial signs of activity. Indicative of a therapeutic window, studies in mice using a BiTE binding to murine EpCAM and murine CD3 demonstrated anti-tumor activity in the absence of damage to EpCAM-expressing normal epithelia [37], [41], [42] .

Here we asked whether T cells engaged by BiTE antibody MT110 are a means for complete elimination of EpCAM-expressing TICs under cell culture conditions, and in immunodeficient mice. Primary colorectal TICs isolated from human colorectal tumor sections and a subpopulation of *in vivo*-selected, highly tumorigenic HT-29 colorectal cancer cells were here used as source of TICs, and characterized for their tumorigenic potential and other TIC-associated properties. By analysis of *in vitro* cytotoxicity reactions with peripheral human T cells and MT110 in soft agar for potentially surviving target cells, we identified conditions allowing lysis of apparently all TICs. In mice, low doses of MT110 prevented outgrowth of tumors from a 5,000-fold excess of a minimally tumorigenic TIC dose, and all mice treated with the two highest MT110 doses tested survived s.c. inoculation of a highly tumorigenic cell bolus.

## Materials and Methods

### Tissue digestion and cell preparation

Human colorectal tumor sections were procured after patients' written consent under an IRB study for research use of their tissues. Tumor tissue (stage 4) had been collected from three patients who had undergone chemo plus radiation therapy under guidance of the Institutional Review Board from hospitals in the Los Angeles area.

Tumor tissues were initially minced and then digested by collagenase A (0.2 mg/ml) and trypsin (0.05 mg/ml) for 15 minutes at 37°C by gentle pipetting. The cell suspension was centrifuged at 100×g for 7 minutes and the cell pellet washed with complete growth medium (Celprogen, M36112-39PS) for three times. The cells were reconstituted in un-differentiation medium (Celprogen, M36112-39US) and cultured in pre-coated flasks with a chemically defined matrix that selects for CD133-positive cells. For the present study, primary colorectal tumor-initiating cells (TICs) from one 40 year-old male colorectal cancer patient (stage 4) were selected because of their high degree of previous characterization and expandability in cell culture.

Colon tumor xenografts derived from human CRC line HT-29 cells were removed from NOD/SCID mice and minced into 1 mm^3^ pieces. After one washing step in PBS, tumor tissues were incubated in digestion buffer of the Cancer Cell Isolation Kit (Panomics) for 1–2 h at 37°C with mechanical disruption every 30 min. Single cells were obtained through a 100 µm cell strainer (BD Falcon), and were separated from hematopoietic and dead cells by gently loading the cell suspension onto a layer of purification buffer from the Cancer Cell Isolation Kit (Panomics). Sedimented tumor cells were washed twice in PBS and checked for vitality using trypan blue exclusion assay.

Lineage-positive host cells were depleted from tumor cells using the MACS Lineage Cell Depletion Kit (Miltenyi Biotec). Anti-mouse CD45 (eBioscience) and anti-mouse CD29 (eBioscience) antibodies were used to test for the presence of contaminating mouse cells.

### Cell culture and animals

TICs were cultured in NS-A basal serum-free medium (Euroclone) containing 2 mM L-glutamine, 0.6% glucose, 9.6 µg/ml putrescine, 6.3 ng/ml progesterone, 5.2 ng/ml sodium selenite, 0.025 mg/ml insulin, 0.1 mg/ml transferrin sodium salt, supplemented with 20 ng/ml EGF and 10 ng/ml basic FGF. HT-29 cells were purchased from the American Type Culture Collection and maintained in RPMI medium (Biochrom) containing 10% fetal bovine serum (Biochrom).

NOD/SCID mice were obtained from the Jackson Laboratory (Charles River). All experiments involving animals were performed in accordance with the relevant guidelines for the care and use of animals and with approval by the responsible animal welfare authority, the Regierung von Oberbayern (Appoval ID: 55.2-1-54-2531.2-12-09).

### Genomic DNA sequencing

Genomic DNA was extracted from cells using the DNeasy Blood and Tissue Kit (Qiagen) following manufacturer's instructions. Exon 9 and 20 (codons 1023 and 1047) of *PIK3CA*, exon 2 of *KRAS* and exon 15 of *BRAF* were amplified with primers complementary to surrounding sequences. PCR products were purified with the QIAquick Gel Extraction Kit (Qiagen) and sequenced. Sequences have been deposited in GenBank (accession number BankIt1358613 HM459602, HM459603, HM459604, HM459605).

### Magnetic bead isolation

CD44-positive cells derived from HT-29 xenograft tumors were sorted using the Pan anti-Mouse Kit (Invitrogen) according to manufacturer's instructions. Briefly, cells were incubated 30 min with magnetic beads pre-coated with anti-CD44 antibody (Chemicon). CD44^high^ and CD44^low^ cells were seperated by a Dynal magnet. CD44^high^ bead-bound cells were washed three times with PBS plus 2% FCS and incubated with DNaseI to release the cells from the beads via DNA linker cleavage. The flow-through was incubated again with new CD44 antibody-coated magnetic beads to obtain a pure CD44^low^ fraction.

### Flow cytometry

Cells were trypsinized, washed with PBS containing 2% FCS and stained with 5–10 µg/ml of the following antibodies for 30 min at 4°C: CD44-PE (Chemicon), CD24-APC (Immunostep), CD44v6-FITC (Abcam), CD133/2-PE (Miltenyi), CD166-ALEXA 647 (Serotec), ABCG2-PE (eBioscience), E-Cadherin (SantaCruz) and EpCAM (MT110, Micromet AG). For analysis a FACSCalibur flow cytometer (Beckton Dickinson) was used.

### Redirected lysis and colony formation assay

Ten thousand to 20,000 TICs were incubated with unstimulated human peripheral blood mononuclear cells (PBMCs) or PBMCs depleted of CD4^+^ T cells, CD56^+^ NK and NKT cells (i.e. enriched for CD8^+^ T cells) [29] at an E:T ratio of 1∶5-10∶1 and different antibody concentrations ranging from 0.01–200 ng/ml. After incubation for 20–120 h, a soft-agar colony assay was performed. To this end, supernatant and trypsinized cells were suspended in RPMI medium containing 10% FCS and 0.4% agarose and subsequently overlayed onto a solidified layer of RPMI medium containing 10% FCS and 0.5% agarose. After 11–17 days, colony forming units (CFU) with >70 µm in diameter were counted. For photographs, CFU were stained with 0.05% crystal violet (Sigma).

### Transplantation of TICs or CFU

Cells were trypsinized, washed once with PBS, resuspended in serum-free medium, mixed with matrigel (BD Biosciences) 3∶1 and injected s.c. into flanks of 6–8 weeks old female NOD/SCID mice (Charles River Laboratories).

For transplantation of CFU, 1 or 10 colonies, >70 µm in diameter were picked with a micromanipulator (CellTram Oil, Eppendorf). Colonies were immediately injected s.c. into NOD/SCID mice. All animals were monitored once a week for tumor formation. Growing tumors were measured with a caliper and tumor volumes were calculated according to the formula: tumor volume  =  [(width^2^*length)/2].

Animals injected with colonies were generally sacrificed after 5–6 months and examined for lymph node metastasis. Metastases were dissected, digested and analyzed by FACS using anti-human CD24, CD44, EpCAM, and anti-murine CD45 antibodies.

### Efficacy analysis of MT110 in TIC xenograft models

1×10^6^ human PBMCs isolated from heparinized fresh whole blood of a healthy donor were mixed with 5×10^5^ TICs in a final volume of 200 µl. The PBMC effector/target cell mixture (E:T of 2∶1) was s.c. injected into the right flank of each NOD/SCID mouse. At least 3 animals per group were intravenously treated with MT110 or PBS control vehicle starting 2 h after inoculation with the indicated doses. MT110 was administered daily for a total of 12 days.

In the HT-29 model mixtures of 50,000 highly tumorigenic HT-29 xenograft-derived CD44^high^/CD24^high^/EpCAM^high^ cells and 1×10^5^ human PBMCs were inoculated into 5 NOD/SCID mice per group. Mice were daily treated i.v. for 12 days with the indicated doses of MT110 or with vehicle controls in the presence of PBMCs.

For elimination of established tumors in NOD/SCID mice by treatment with MT110 mixtures of 5×10^6^ TICs and 1×10^7^ human PBMCs were inoculated into 5 NOD/SCID mice per group to allow solid tumor formation. After tumor establishment at day 4, mice were treated i.v. for 14 days with 2.5 mg/kg of MT110, or with vehicle control in presence of PBMCs.

## Results

### *In vivo*-selected, highly tumorigenic cells of colorectal cancer line HT-29 can be eliminated by MT110-engaged T cells

We selected a highly tumorigenic subpopulation of human HT-29 colorectal cancer cells from tumors growing under the skin of immunodeficient mice. Cells of the colorectal cancer line HT-29 have a mutated *BRAF* gene (V600E) and are relatively resistant to cytotoxicity by anti-EGFR antibody cetuximab [43]. We found that HT-29 cells also had a mutation in the *PIK3CA* gene (E545A). Tumor cells isolated from subcutaneously growing HT-29 tumors were sorted by CD44 and separated into CD44^high^/CD24^high^/EpCAM^high^ and CD44^low^/CD24^low^/EpCAM^low^ subgroups (Figure S1A). EpCAM expression tracked with CD44 expression and was highest in the CD44^high^/CD24^high^ subpopulation. Both isolated subpopulations were essentially free of murine cells as detected by the absence of murine CD29 and CD45 expression (Figure S1B, Materials and Methods S1).

Compared to CD44^low^/CD24^low^/EpCAM^low^ cells, CD44^high^/CD24^high^/EpCAM^high^ cells had higher functional levels of aldehyde dehydrogenase (ALDH) (Figure S1C, Materials and Methods S1), a 30-fold higher tendency to form spheres in cell culture, and a 20-fold higher tendency for colony formation in soft agar (Figure S1D), and higher expression levels of stem cell markers CD133, CD166, the CD44 splice variant v6 and the ABC transporter G2 (Figure S2, Materials and Methods S1). While at least 1×10^5^ non-selected HT-29 cells were required to form tumors in immunodeficient mice (Table 1), minimally 10 CD44^high^/CD24^high^/EpCAM^high^ cells or 100 CD44^low^/CD24^low^/EpCAM^low^ cells were sufficient for inducing a tumor in mice in two independent experiments. This showed that we had isolated highly tumorigenic subpopulations of cells from xenografts with the fraction of CD44^high^/CD24^high^/EpCAM^high^ cells having the most pronounced TIC phenotype.

**Table 1:** Tumorigenicity of HT-29 colorectal cancer cell populations and primary colorectal TICs.

Cell Type	Number Injected	TumorIncidence	Latency[Days]	LymphNodeMetastasis
HT-29 Unsorted	1001,00010,000100,0001,000,000	0/20/20/22/22/2	>180>180>18084	n.d.
HT-29 CD44^low^/CD24^low^/EpCAM^low^	101001,000	0/22/22/2	>1803333	n.d.
HT-29 CD44^high^/CD24^high^/EpCAM^high^	101001,000	2/22/22/2	333333	n.d.
TICs	101001,00010,000100,0001,000,000	0/24/44/44/44/42/2	>8042–6637–7821–3010–2110–21	n.d.
Soft Agar only		0/2	>100	0
Single TIC Colony	1	3/6	24–60	2/6
Mix of TIC Colonies	10	2/6	53–59	1/6

Subpopulations of human HT-29 cells were isolated from subcutaneously growing tumors of mice based on CD44 and CD24 expression levels. Tumorigenicity was tested by subcutaneous injection of the indicated number of tumor cells or soft agar colonies derived from TICs into NOD/SCID mice.

**Figure 1:**
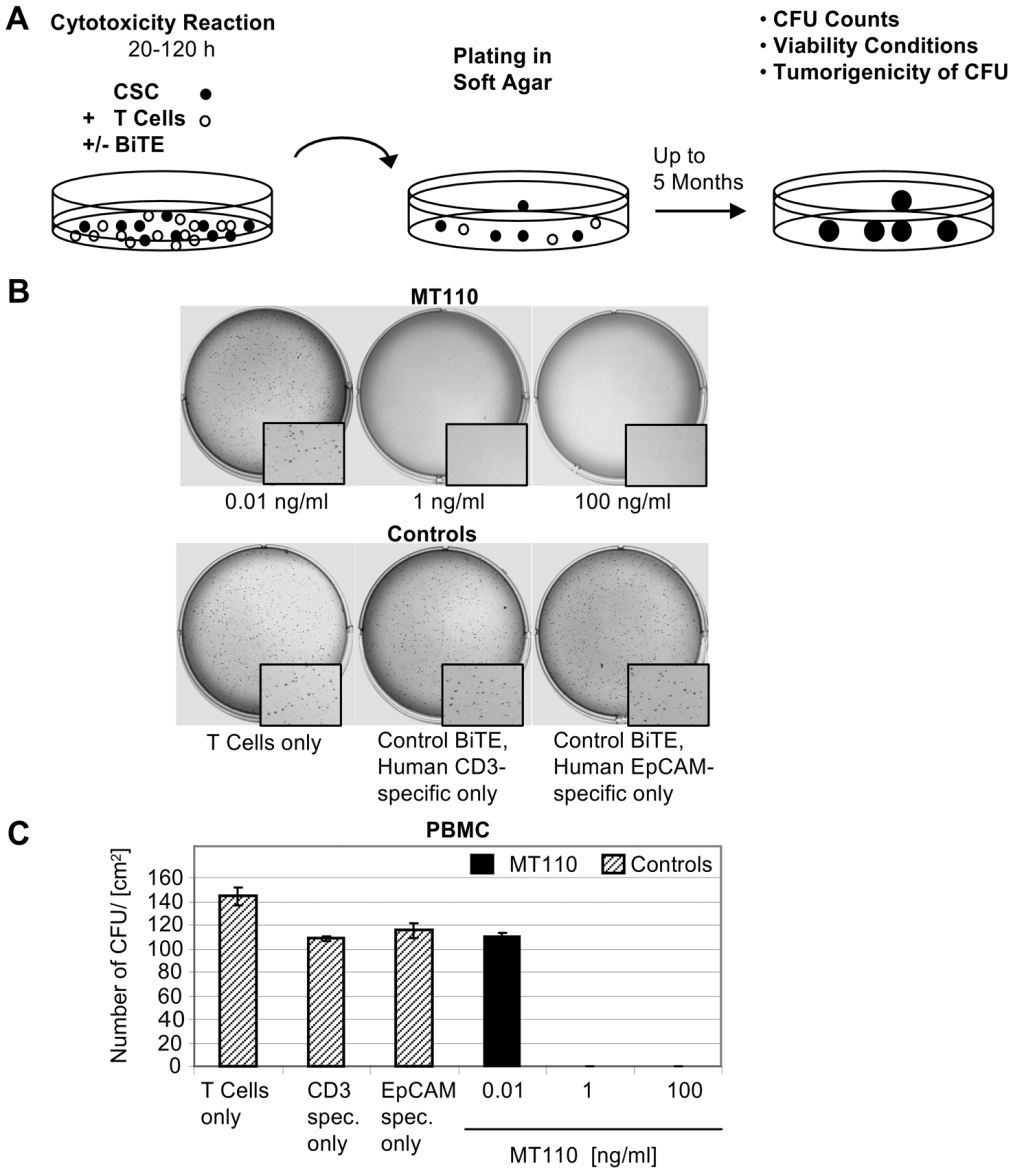
Eradication of tumor-initiating CD44^high^/CD24^high^/EpCAM^high^ HT-29 colorectal cells by MT110-engaged T cells. (A) Scheme of cytotoxicity assay with soft agar-based assay readout. (B) Photographs of soft agar plates with crystal violet-stained colonies growing from CD44^high^/CD24^high^/EpCAM^high^ cells treated in the presence of T cells at the indicated concentrations of MT110, control BiTE antibodies at 100 ng/ml, or T cells alone. (C) Effect of MT110 on the number of CFUs per cm^2^. Bar graphs are mean values after 14 days from triplicate determinations +/− SD. Unstimulated PBMC were used as effector T cell source at an PBMC to HT-29 cell ratio of 10∶1.

In order to investigate the efficacy of MT110-engaged T cells in redirected lysis of CD44^high^/CD24^high^/EpCAM^high^ HT-29 cells, *in vitro* cytotoxicity reactions were performed using unstimulated peripheral blood mononuclear cells (PBMCs) as source of T cells at an effector to target (E:T) ratio of 10∶1. Subsequently, cell culture reactions were mixed with soft agar. This way, cancer cells potentially surviving the BiTE reaction in the presence of T cells can be detected with high sensitivity and quantified by formation of colonies. The assay procedure is schematically shown in Figure 1A. One (18 pM) or 100 ng/ml MT110 were sufficient to completely eliminate tumorigenic HT-29 cells while 0.01 ng/ml MT110, PBMC alone, or control BiTE antibodies solely binding CD3 or EpCAM had no effect on the number of colonies formed (Fig. 1B). A total of 20,000 CD44^high^/CD24^high^/EpCAM^high^ HT-29 cells were used per cytotoxicity reaction, which under control conditions gave between 110 and 145 visible colonies per cm^2^ after 14 days (Fig. 1C). In a standard cytotoxicity assay, the half maximal concentration for redirected lysis of HT-29 bulk, CD44^high^/CD24^high^/EpCAM^high^ and CD44^low^/CD24^low^/EpCAM^low^ subgroups was determined at 1.9 +/− 0.6 ng/ml, 0.05 +/− 0.05 ng/ml and 1.5 +/− 0.1 ng/ml of MT110, respectively (Figure S3A, Materials and Methods S1).

### Characteristics of primary colorectal TICs

We next investigated colorectal TICs derived from tumor sections of CRC patients. In order to verify their cancer stem cell phenotype, TICs were analyzed by FACS staining for expression of established surface markers [18], and by using RT-PCR for expression of mRNAs coding for surface and intracellular marker proteins. FACS staining showed that TICs were CD44^high^/CD24^high^/EpCAM^high^, and expressed CD133, CD166, the CD44 splice variant v6 and the ABC transporter G2 on their surface (Figure S3A, Materials and Methods S1). RT-PCR analysis verified the expression of the surface antigens at the mRNA level. Furthermore, robust RT-PCR signals were detected for alkaline phosphatase, CA199, nestin, notch-1, Oct-4, SSEA 3/4 and telomerase. Genomic sequence analysis showed that primary colorectal TICs had mutations in KRAS (G13D) and PI3 kinase genes (E545K, E545T, and D549N, data not shown).

We found that a dose of 100 TICs was minimally required for induction of tumors in NOD/SCID mice with a latency of 42–66 days (Table 1). Higher cell doses showed increasingly shorter latency periods. Ten TICs were not sufficient to induce a tumor during an observation period of 80 days. When TICs were grown in cell culture, they had a tendency to form dense spheres of 100 µm in diameter and larger (Figure S4A). Under cell culture conditions in the presence of 10% fetal calf serum (FCS), TICs started to differentiate as shown by expression of E-cadherin [44] and a morphological change to a more epithelial phenotype (Figure S4B, Materials and Methods S1). In NOD/SCID mice, 10,000 TICs grown in TIC medium were more tumorigenic in comparison to TICs cultured under serum containing conditions (data not shown). The percentage of cells retaining their PKH26-membrane label declined over time upon the change to the FCS-containing medium assuming an increase in symmetric cell division and loss of self-renewal capacity (Figure S4C, Materials and Methods S1). When inoculated in mice, xenografts from TICs after 4 weeks contained between 0.2% and 0.5% PKH-26 labeled cells (Figure S4D, Materials and Methods S1), demonstrating differentiation of most TICs but also self-renewal of a small subset of TICs. The content of TICs in the xenografted tumors was comparable to that found in tumor biopsies of patients. Lastly, 3,000 PKH-26 labeled cells isolated from TIC xenografts formed tumors after re-inoculation in mice (data not shown).

In conclusion, expression of a specific set of marker proteins, high tumorigenicity, differentiation in growth factor-containing medium and within xenografts, formation of spheres, and self-renewal capacity suggest that the colorectal TICs used in this study behaved like *bona fide* tumor-initiating cells and were therefore valuable as EpCAM-expressing TICs for testing the therapeutic potential of BiTE antibody MT110 *in vitro* and *in vivo*.

### Activity of MT110-redirected T cells against TICs *in vitro*

**Figure 2:**
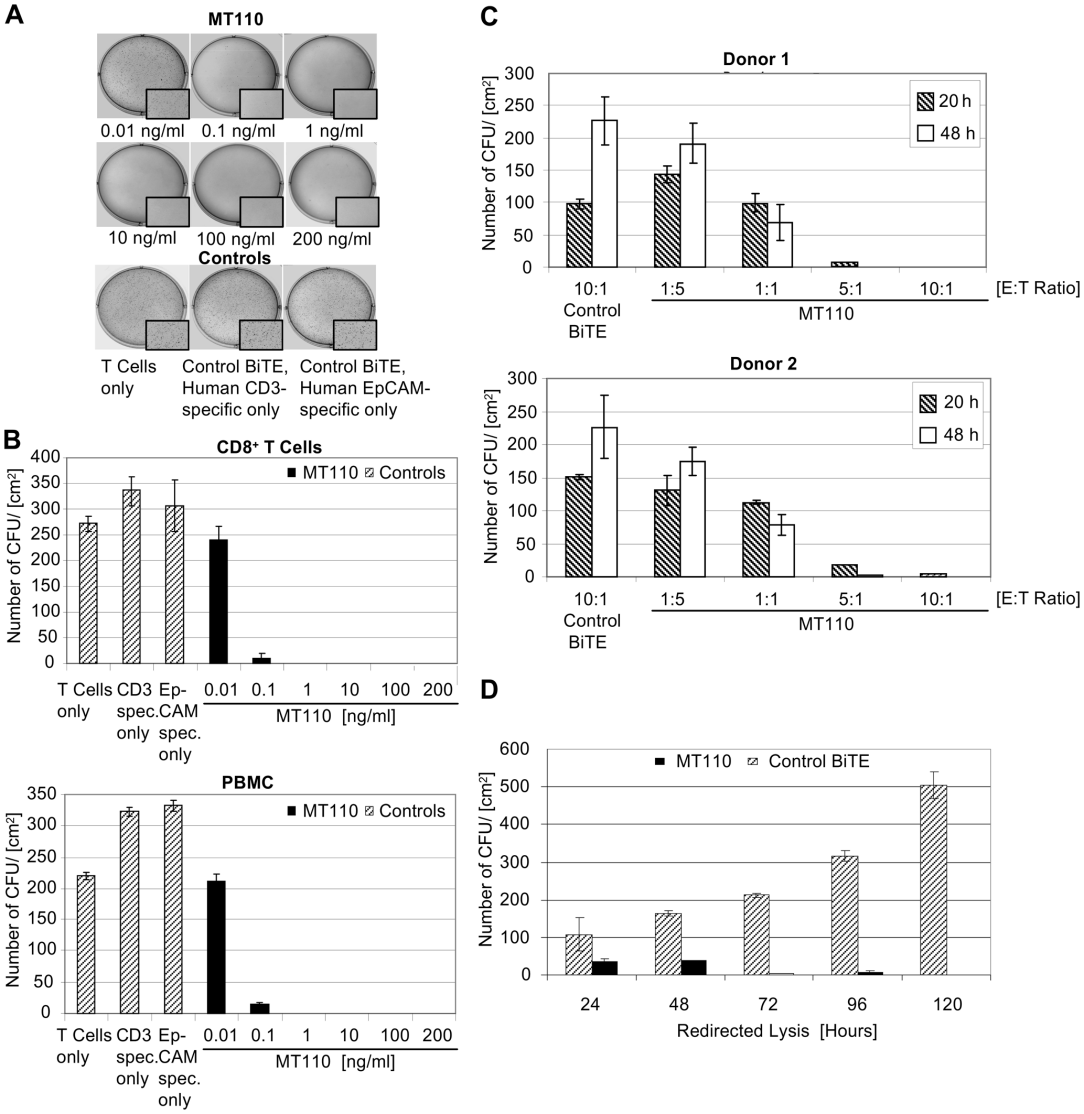
Eradication of primary colorectal tumor-initiating cells (TICs) by MT110-engaged T cells. (A) Photographs of crystal violet-stained colonies growing from TICs treated in the presence of T cells with the indicated concentrations of MT110, control BiTE antibodies at 200 ng/ml, or T cells alone. Unstimulated PBMCs were used as source of T cells at a PBMC to TIC ratio of 1∶1. (B) MT110 dose response of redirected lysis. Number of CFUs from TICs per cm^2^ are shown. Bar graphs are mean values of colony numbers from triplicate determination +/− SD observed after 11 days. Unstimulated PBMCs or CD8^+^ cells were used as effectors as indicated at an E:T ratio of 10∶1. (C) Effect of E:T ratio and T cell donor on redirected lysis. Number of CFUs per cm^2^ of TIC cells treated with 10 ng/ml MT110 or a CD3-monospecific BiTE control antibody at the indicated E:T ratios and incubation times are shown. Bar graphs are mean values of colony numbers from triplicate determination +/− SD as observed after 17 days. Unstimulated PBMCs from two healthy blood donors were used as effector cells. (D) Time dependence of redirected lysis. Number of CFUs per cm^2^ from TICs treated with 10 ng/ml MT110 or a CD3-monospecific BiTE control antibody at a PBMC to TIC ratio of 1∶1 are shown. Bar graphs are mean values from a triplicate determination +/− SD observed after 15 days.

Redirected lysis of TICs by EpCAM-specific BiTE antibody MT110 and T cells was investigated by using either PBMCs or CD8^+^ T cells isolated from PBMCs as effector cells. Ten to 20,000 TICs were used per assay, which, under control conditions, gave raise to >200 colony forming units (CFUs) per cm^2^ in soft agar with a diameter of >70 µm after 2 weeks. We first determined the minimal concentration of MT110 required for complete lysis of TICs using CD8^+^ T cells or PBMCs as effectors. An E:T ratio of 10∶1 was used and the cytotoxicity reaction allowed for 20 h. A very substantial decrease in CFU formation was already seen with a concentration of 0.1 ng/ml MT110, i.e., 2 pM of the bispecific antibody (Fig. 2A and 2B). Cytotoxicity reactions testing MT110 concentrations of 1, 10, 100 and 200 ng/ml showed no formation of CFUs when analyzed in soft agar assays. In a standard cytotoxicity assay, the half maximal concentration for redirected lysis of TICs was determined at 0.41 +/− 0.25 ng/ml of MT110 (Figure S3B, Materials and Methods S1). Results were not different if purified CD8^+^ T cells (Fig. 2A and 2B, top) or PBMCs (Fig. 2B, bottom) were tested, indicating that CD8^+^ T cells made a major contribution to redirected lysis and that other T lymphocytes within PBMCs, such as regulatory T cells, did not detectably influence lysis of TICs. The number of CFUs obtained in the presence of human T cells was not affected by two control BiTE antibodies at concentrations of 200 ng/ml that either solely bound to CD3 on T cells or to EpCAM on TICs by using the same single-chain antibody arms as in MT110.

In order to demonstrate that lysis of TICs was dependent on immune cells and was similar with immune cells from different human PBMC donors, four E:T ratios of 1∶5, 1∶1, 5∶1 and 10∶1 were tested for efficacy with PBMC from two human donors in cytotoxicity reactions for 20 or 48 h using 10 ng/ml MT110. PBMCs typically contain 15–20% CD8^+^ T cells, indicating that effective CD8^+^ T cell to target cell ratios would be at least 5-fold lower than for PBMCs. While an E:T ratio of 1∶1 gave only a partial reduction of CFUs after 20 h, redirected lysis at E:T ratios of 5∶1 and 10∶1 almost completely prevented CFU formation in soft agar (Fig. 2C). With PBMCs from donor 1 at an E:T ratio of 10∶1, no detectable CFUs had formed after 17 days in soft agar.

We next analyzed the time dependence of TIC lysis. Compared to a control BiTE solely binding CD3, 10 ng/ml of EpCAM/CD3-bispecific BiTE antibody MT110 led to a pronounced reduction of the number of CFUs after a 24 h-cytotoxicity reaction at a low E:T ratio of PBMC to TICs of 1∶1 (Fig. 2D). Approximately 5 CFU/cm^2^ were detected after cytotoxicity reactions for 72 and 96 h, and none was seen if redirected lysis was allowed for 120 h. Under control conditions, CFU numbers steadily increased over time showing that TICs well proliferated in the presence of PBMCs and the absence of MT110.

Under conditions where no CFUs were observed in soft agar after 11–17 days, CFU formation in some cases was monitored for up to 5 months. In no single case, CFUs were seen after this long observation period. Figure S5 shows that soft agar conditions after 3 and 5 months still supported the outgrowth of colonies when TICs were transferred into the aged agar. We therefore conclude that under conditions where no CFU formation was observed, all TICs had been lysed by MT110-redirected T cells in the preceeding cytotoxicity reaction.

### TICs growing in soft agar are highly tumorigenic and form metastases

**Figure 3:**
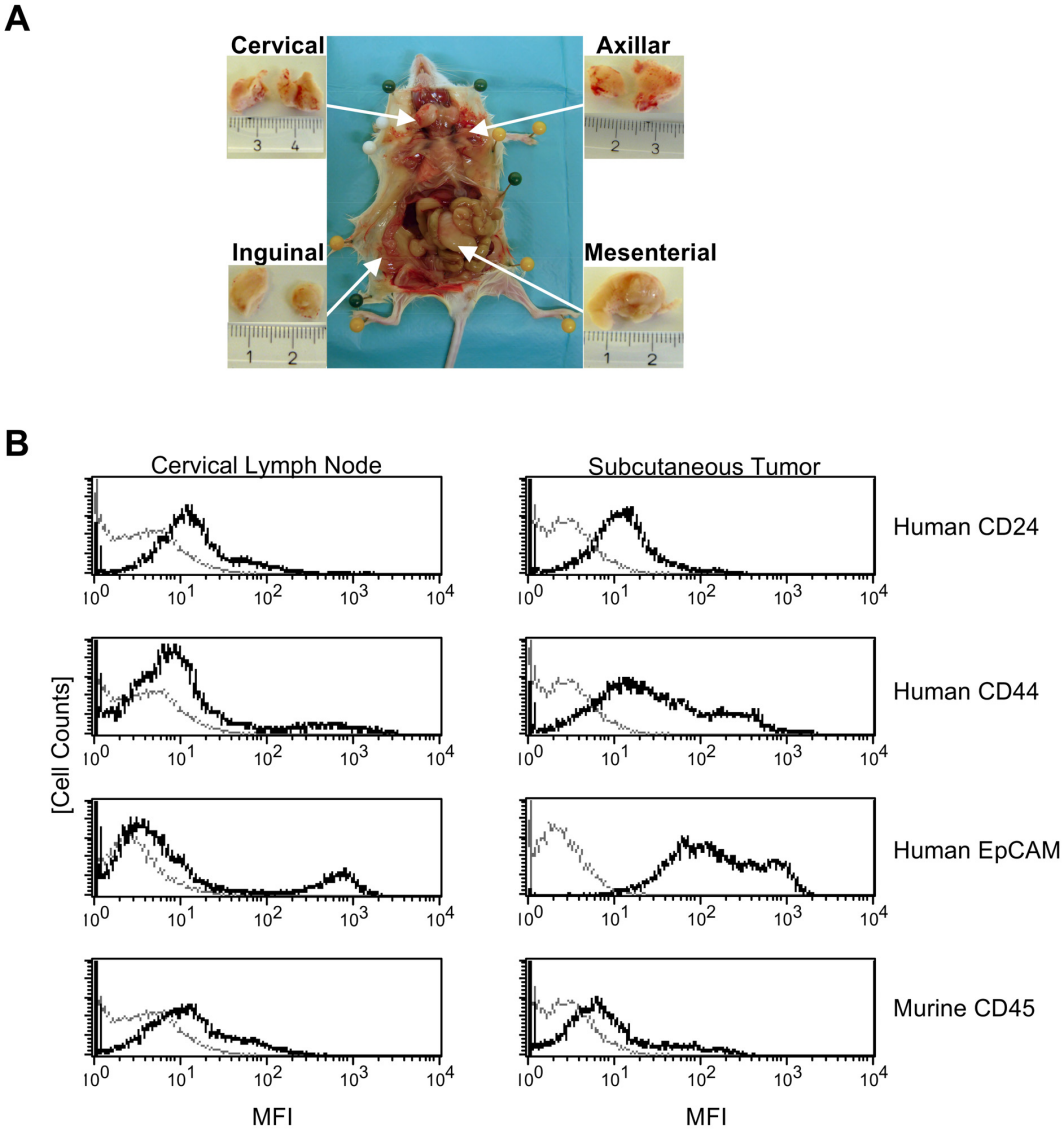
Soft agar colonies from TICs implanted into NOD/SCID mice cause lymph node metastasis. (A) Representative photographs of lymph node metastases derived from s.c. implanted TIC-derived soft agar colonies. (B) Representative FACS analysis of cells derived from an s.c. tumor and a cervical lymph node of a NOD/SCID mouse s.c. implanted with a soft agar colony derived from TICs.

We further investigated whether CFUs formed in soft agar still contain highly tumorigenic TICs. Single colonies (1 CFU≈200–300 cells) or mixes of 10 colonies were isolated from soft agar plates and subcutaneously injected into NOD/SCID mice. While soft agar alone did not cause tumors, tumor growth from single colonies was observed in 50% of cases with a latency period of 24–60 days, and in 33% of cases after 53–59 days for mixes of colonies (Table 1). CFUs did not only form local subcutaneous tumors but gave rise to extensive lymph node metastasis. When tumor-bearing mice were sacrificed and inspected, enlarged lymph nodes were found in various locations (Fig. 3A). Cellular analysis of lymph node homogenates by FACS staining revealed that they contained cells expressing human CD24, CD44 and EpCAM (Fig. 3B, left). The same human marker proteins were found on cells from subcutaneous TIC-derived tumors (Fig. 3B, right).

### Activity of MT110 against TIC-derived tumors in a mouse model

**Figure 4:**
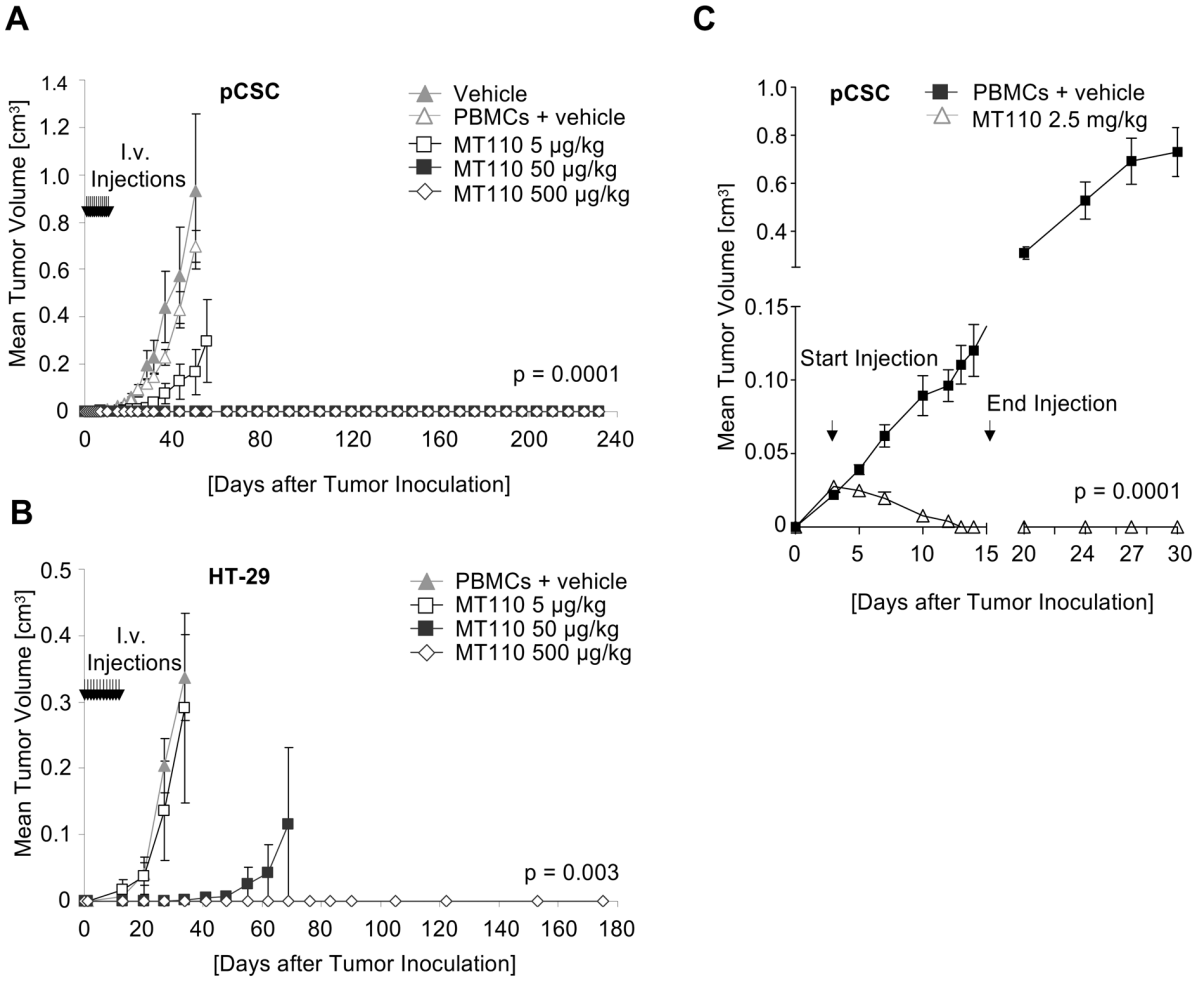
Cure of NOD/SCID mice receiving a 5,000-fold excess of a minimally tumor-initiating TIC-dose. (A) Mixtures of 5×10^5^ TICs and 1×10^6^ human PBMCs were inoculated into NOD/SCID mice (N = 3 for vehicle; N = 5 for vehicle + PBMCs; N = 4 for MT110 5 µg/kg; N = 3 for MT110 50 µg/kg and N = 3 for MT110 500 µg/kg group) and mice daily treated i.v. for 12 days with the indicated doses of MT110, or with vehicle controls in the absence and presence of PBMCs. The mean tumor volume is given. P-value was determined by Student's t-test. (B) Mixtures of 50,000 highly tumorigenic HT-29 xenograft-derived CD44^high^/CD24^high^/EpCAM^high^ cells and 1×10^5^ human PBMCs were inoculated into 5 NOD/SCID mice per group and mice daily treated i.v. for 12 days with the indicated doses of MT110, or with vehicle controls in the presence of PBMCs. The mean tumor volume is given. P-value was determined by Student's t-test. (C) Elimination of established tumors in NOD/SCID mice by treatment with MT110. Mixtures of 5×10^6^ TICs and 1×10^7^ human PBMCs were inoculated into 5 NOD/SCID mice per group to allow solid tumor formation. After tumor establishment at day 4 mice were treated i.v. for 14 days with 2.5 mg/kg of MT110, or with vehicle control in presence of PBMCs. The mean tumor volume is given. P-value was determined by Student's t-test.

Initial experiments had shown that 100 TICs or 10 HT-29 CD44^high^/CD24^high^/EpCAM^high^ cells were sufficient to induce tumors in NOD/SCID mice with a latency of 42–66 and 33 days, respectively (Table 1). In order to test the *in vivo*-efficacy of MT110 against TICs and HT-29 CD44^high^/CD24^high^/EpCAM^high^ cells, we established a mouse model where tumor growth is induced by a 5,000-fold higher dose of colorectal TICs as is minimally needed for tumor initiation (Fig. 4). As shown in Figure 4A, 0.5×10^6^ TICs caused tumors growing to a size of >0.6 cm^3^ after 50 days, with tumors detectable before day 20. The presence of human PBMCs at an E:T ratio of 2∶1 in subcutaneous tumors, as is needed by MT110 for redirected lysis in immunodeficient mice, did not significantly retard or reduce tumor growth. Doses of 50 or 500 µg/kg MT110 given intravenously for the first 12 days completely prevented tumor formation for the entire observation period of 232 days, and all mice survived inoculation of the high TIC number (Fig. 4A). MT110 at 5 µg/kg also significantly inhibited tumor outgrowth, and 50% of mice survived of which one developed no tumor, one remained static at a tumor volume of 16 mm^3^ for 71 days but eventually became free of tumor, and two had to be sacrificed with large tumors for welfare reasons. In the vehicle arm, all mice had to be sacrificed for welfare reasons 55 days after start of the experiment.

Similar results were obtained with the HT-29 CD44^high^/CD24^high^/EpCAM^high^ tumor model (Fig. 4B). Doses of 500 µg/kg MT110 i.v. for 12 days prevented tumor outgrowth in 5 out of 5 mice for the entire observation period of 180 days. At a dose of 50 µg MT110/kg per day, 4 out of 5 mice were free of tumor and at a dose of 5 µg MT110/kg per day, 1 out of 5 mice was tumor-free. This shows that T cells engaged by MT110 are capable of eliminating also in animals an excessive dose of highly tumorigenic TICs.

MT110 was also efficient in treatment of established tumors derived from colorectal TICs (Fig. 4C). At a dose of 2.5 mg MT110/kg given daily i.v. for 14 days, a complete remission of tumors in 5 out of 5 mice until the end of the study at day 30 was observed (Fig. 4C).

## Discussion

We here show that cytotoxic human T cells engaged by extremely low amounts of an EpCAM/CD3-bispecific antibody construct, which is currently in a phase I trial, have the potential to completely eradicate two kinds of highly tumorigenic colorectal tumor-initiating cells. One kind was *in vivo*-selected CD44^high^/CD24^high^/EpCAM^high^ HT-29 cells, the other primary TICs isolated from the tumor of a CRC patient. The two cell types showed all known hallmarks of TICs, including tumor growth from very low cell numbers, sphere formation, expression of a panel of markers and, in one case, of ALDH activity. Both TIC cell types had mutations in either *BRAF* or *KRAS* and both in *PIK3CA* genes that may render patients insensitive to treatment with signal inhibitory anti-EGFR therapeutics [45], [46]. We here show that such mutated colorectal cancer cells are highly sensitive to lysis by an EpCAM-specific T cell-engaging antibody, suggesting that redirected lysis by T cells is not affected by mutations in intracellular signaling proteins of the EGFR pathway. This is explained by the mode of BiTE antibody action in which the surface target antigen serves as mere anchor for T cell engagement. The highly sub-saturating amounts of BiTE antibody as used for redirected lysis are moreover unlikely to modulate signaling activity of the target in a meaningful way. By contrast, anti-receptor antibodies like panitumumab and cetuximab rely on saturating target binding for signal inhibition.

Complete elimination of cancer-initiating cells by MT110-engaged T cells was demonstrated *in vitro* by a highly sensitive assay using soft agar colony growth, and *in vivo* in a xenograft mouse model testing survival and tumor growth after inoculation of a high TIC bolus. This high anti-tumor activity of MT110 will depend on the expression of the target antigen EpCAM and equally hit colorectal TICs and derived bulk tumor cells. Colorectal cancers show the most frequent EpCAM expression of all human adenocarcinoma analyzed to date [12] with 99.7% of primary tumor samples (N = 1,186 CRC patients) being positive. Moreover, independent reports showed expression of EpCAM on colorectal TICs [5], [9], [47], [48], with evidence for a functional role of EpCAM in tumorigenesis [5], [20]. Involvement of EpCAM in *wnt* signaling [22] may ensure that a majority of TICs need to express EpCAM for proliferation and survival and that target loss may select a population of tumor cells with reduced tumorigenic potential.

In our xenograft model, unstimulated human PBMCs were mixed with TICs prior to subcutaneous implantation. In the absence of BiTE antibody, this pre-mixing of cells will have no biological consequence for T or tumor cells. Only when intravenously infused BiTE antibody MT110 is reaching tumor tissue in the skin, local T cells can become activated and eventually lyse tumor cells. A recent study has shown that subcutaneous tumors derived from a pancreas cancer cell line, which were grown up to 300 mm^3^ in the absence of human T cells, can be eradicated by intraperitoneally administered human T cells in the presence of intravenously administered BiTE antibody MT110 [49]. This suggests that BiTE antibodies do not necessarily rely on tumor infiltrated T cells but can likewise engage peripheral T cells for tumor rejection.

T cells may be particularly suited to eliminate disseminated TICs. Effector memory T cells are constantly roaming our organs in search for target cells, and are readily activated upon rechallenge. Moreover, in colorectal cancer the presence of T cells in tumors correlates with survival of patients [50]. We have shown in clinical [40] and preclinical studies [51] that effector memory T cells make the largest contribution to BiTE activity, and that redirected T cells can lyse tumor cells independent of T cell receptor specificity, MHC class I expression and the need for costimulation [30], [34], [35]. By their search and destroy mode of action and their high cytotoxic potential [30], [31], T cells may be able to find widely disseminated, temporarily non-proliferating TICs. The elimination of therapy-resistant TICs or circulating tumor cells by antibody-engaged T cells may improve the outcome of standard therapies and reduce mortality of cancer patients.

## Supporting Information

Materials and Methods S1 Supplementary Materials and Methods.
(0.03 MB DOC)

Figure S1 Stem cell features of colorectal HT-29 cancer cells trace with CD44^high/^CD24^high/^EpCAM^high^ phenotype. FACS analysis of human HT-29 cells isolated from mouse xenografts by (A) their levels of CD44 and CD24 expression, (B) expression of human EpCAM, murine CD29 and CD45, viability using nuclear dye propidium iodide, and (C) expression of ALDH by Aldefluor® staining in the presence or absence of ALDH inhibitor diethylamino-benzaldehyde (DEAB). The x-axis shows mean fluorescence intensity (MFI); the y-axis MFI for CD24 expression (A), cell counts (B), or sideward scatter (C). (D) Sphere formation and soft agar colony growth of CD44^high/^CD24^high/^EpCAM^high^ and CD44^low/^CD24^low/^EpCAM^low^ HT-29 cancer cells. Spheres and crystal violet-stained soft agar colonies were counted after 18 days. The mean number of spheres formed per 96-well plate is shown +/- standard deviation (SD), as well as the mean number of colony forming units (CFUs) +/- SD per cm2 from a triplicate determination.
(6.16 MB TIF)

Figure S2 Enhanced expression of cancer stem cell markers in colorectal HT-29 CD44^high/^CD24^high/^EpCAM^high^ cells. CD44^high/^CD24^high/^EpCAM^high^ and CD44^low/^CD24^low/^EpCAM^low^ HT-29 cancer cells isolated from mouse xenograft have been analyzed for EpCAM, Β-catenin, ABCG2, CD44v6, CD133 and CD166 by semiquantitative RT-PCR.
(0.54 MB TIF)

Figure S3 Cytotoxicity assays using HT-29 xenograft-derived and primary tumor-initiating target cells. (A) HT-29 xenograft-derived bulk cells and cells sorted for CD44^high/^CD24^high/^EpCAM^high^ and CD44^low/^CD24^low^/EpCAM^low^ were analyzed by ^51^Cr-release standard cytotoxicity assay. (B) TICs were analyzed by ^51^Cr-release standard cytotoxicity assay. CD8-enriched T cells were used at an E:T ratio of 10:1. Results from triplicate determinations are shown as mean values with standard deviations.
(1.65 MB TIF)

Figure S4 Characterization of TICs for their cancer stem cell phenotype. (A) Sphere formation (left panel) and FACS analysis of cancer stem cells for markers by FACS analysis. MFI-values relative to isotype control are shown (right panel). (B) TICs were cultured under serum-free conditions in TIC medium or under differentiation conditions in RPMI supplemented with 10% FCS in collagen-coated culture flasks. Upper panel: FACS staining of E-cadherin (black line) vs. control (grey line) and lower panel: microphotographs of TICs cultured under FCS and TIC growth conditions taken at 40x and 100x magnification. (C) TICs cultured under serum-free conditions (TIC medium) and differentiation conditions (RPMI supplemented with 10% FCS) analyzed for PKH-26 staining by FACS after 48 and 96 h. (D) PKH-26 labeled TICs were engrafted into NOD/SCID mice. After 4 weeks, the retention of PKH-26 label was determined on TICs by FACS after tumor digestion.
(9.70 MB TIF)

Figure S5 Soft agar still supports colony growth after a long time. Colony growth by TICs inoculated into 3- or 5-month old soft agar.
(2.11 MB TIF)
