# Supplementary material for: Highly Efficient Elimination of Colorectal Tumor-Initiating Cells by an EpCAM/CD3-Bispecific Antibody Engaging Human T Cells
Source: PLoS One. 2010 Oct 18;5(10):e13474. doi: 10.1371/journal.pone.0013474 (PMC2956687; doi:10.1371/journal.pone.0013474)
Supplement: Materials and Methods S1 — Supplementary Materials and Methods. (0.03 MB DOC) [file pone.0013474.s001.doc]

**Supplement material and methods**

**Aldefluor assay.** The Aldefluor Kit (StemCell Technologies) was used according to the manufacturer´s instructions to detect cells with a high ALDH enzymatic activity.

**Sphere formation assay.** Five thousand cells were seeded in 96-well culture plates with serum-free medium supplemented with 20 ng/ml EGF and 10 ng/ml FGF. Medium was changed every 72 h and spheres counted after 18 days.

**RNA extraction and reverse transcription–PCR**

Total RNA was isolated from TICs or HT-29 xenograft cells using the RNAeasy Kit (Qiagen) according to the manufacturer’s protocol. One hundred nanograms of purified mRNA was used to synthesize cDNA using the One-Step RT-PCR Kit (Roche).

**Cytotoxicity assay**

PBMCs with prior stimulation by immobilized CD3 antibody (OKT-3, Janssen-Cilag GmbH, Orthoclone, final concentration 1 µg/ml), CD28 antibody (Becton Dickinson, final concentration 1 µg/ml) and IL-2 (final concentration 20 U/ml) were used as effector cells after depletion of CD4 and CD56 positive cells.

Target cells were labeled by incubation with approximately 7 MBq Chromium-51 for 1 h, followed by removal of free chromium with two washing steps.

HT-29 bulk cells, HT-29 sorted for CD44^high^/CD24^high^/EpCAM^high^ and CD44^low^/CD24^low^/EpCAM^low^ and colorectal TICs were used as target cells (1000 target cells/well). Equal volumes of target and effector cell suspension were then mixed, resulting in an effector-to target ratio (E:T) of 10 to 1. Serial BiTE dilutions or complete RPMI medium as a background control were then added and incubated for 18 h at 37°C in 5% CO_2_. Cytotoxicity was measured as relative values of released chromium in the supernatant related to the difference of maximum lysis (determined by addition of Triton-X 100) and spontaneous lysis (in the absence of effector cells). All measurements were carried out in triplicates. Measurement of chromium activity in the supernatants was performed with a Wizard-300 gammacounter (Perkin Elmer Life Sciences GmbH, Cologne, Germany).

**Flow cytometry.** Cells were trypsinized, washed with PBS containing 2% FCS and stained with 5-10 µg/ml of the following antibodies for 30 min at 4°C: CD44-PE (Chemicon), CD24-APC (Immunostep), CD44v6-FITC (Abcam), CD133/2-PE (Miltenyi), CD166-ALEXA 647 (Serotec), ABCG2-PE (eBiosience), E-Cadherin (SantaCruz) and EpCAM (MT110, Micromet AG). A FACSCalibur flow cytometer (Beckton Dickinson) was used.

**PKH-26 staining experiments**

PKH-26 (Sigma) staining was performed according to the manufacturer´s recommendation with TICs cultured in serum-free medium and in medium with 10% FCS.

5x10^4^ PKH26 stained TICs were seeded into 6-wells and cultured under the above mentionend conditions. At the indicated timepoints the viable cell number and the fluorescence of PKH-26 was analyzed by flow cytometry. Shown are representative results of three independent experiments.

For *in vivo* experiments TICs cultured under serum-free conditions were labeled with PKH-26 (Sigma) and 10,000 cells were inoculated into NOD/SCID mice. After 4 weeks tumors were removed, digested as previously described and analyzed by FACS for PKH-26 fluorescence.
