# Supplementary figures and images for: Highly Efficient Elimination of Colorectal Tumor-Initiating Cells by an EpCAM/CD3-Bispecific Antibody Engaging Human T Cells
Source: PLoS One. 2010 Oct 18;5(10):e13474. doi: 10.1371/journal.pone.0013474 (PMC2956687; doi:10.1371/journal.pone.0013474)

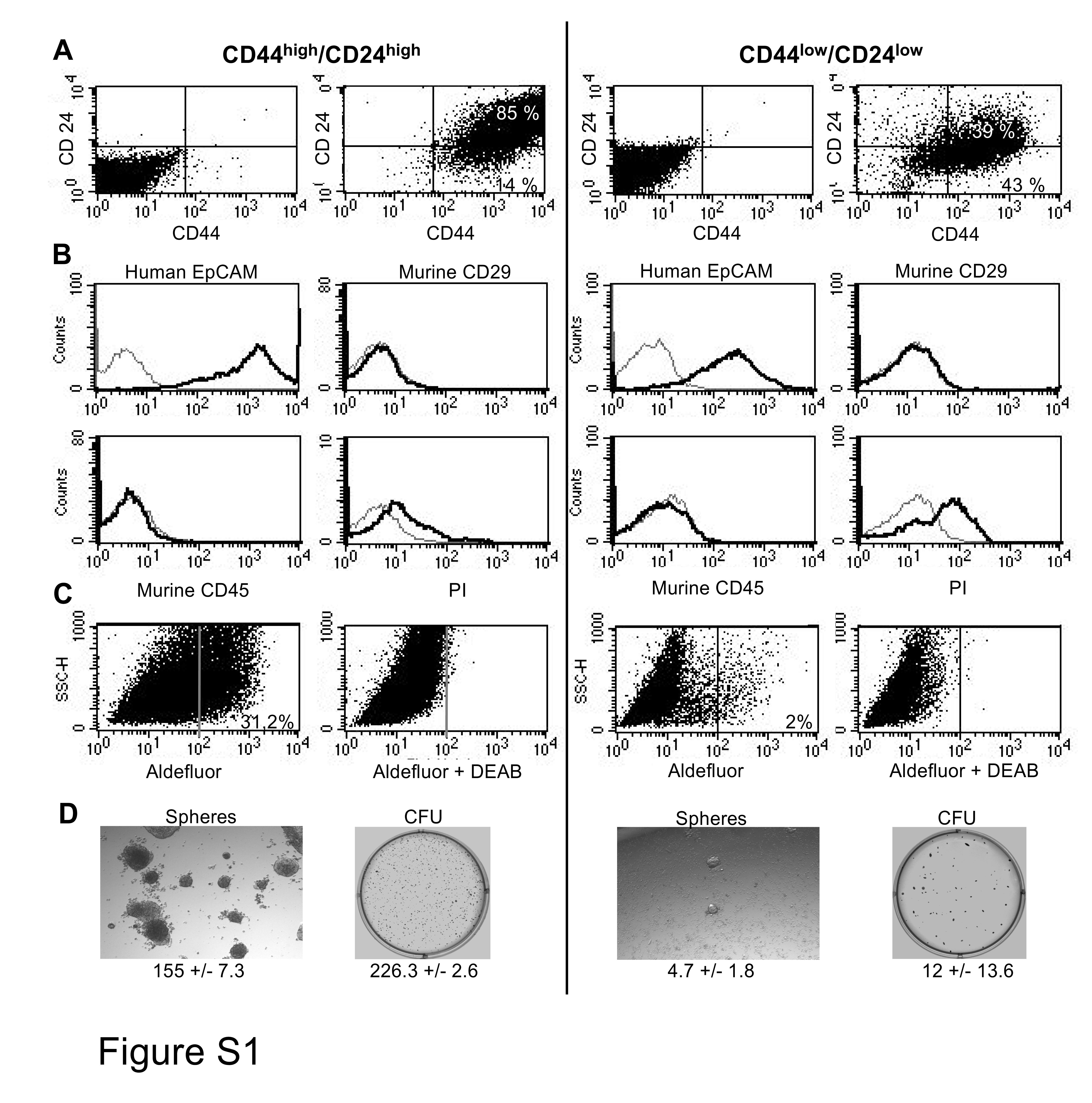

Supplement: Figure S1 — Stem cell features of colorectal HT-29 cancer cells trace with CD44high/CD24high/EpCAMhigh phenotype. FACS analysis of human HT-29 cells isolated from mouse xenografts by (A) their levels of CD44 and CD24 expression, (B) expression of human EpCAM, murine CD29 and CD45, viability using nuclear dye propidium iodide, and (C) expression of ALDH by Aldefluor® staining in the presence or absence of ALDH inhibitor diethylamino-benzaldehyde (DEAB). The x-axis shows mean fluorescence intensity (MFI); the y-axis MFI for CD24 expression (A), cell counts (B), or sideward scatter (C). (D) Sphere formation and soft agar colony growth of CD44high/CD24high/EpCAMhigh and CD44low/CD24low/EpCAMlow HT-29 cancer cells. Spheres and crystal violet-stained soft agar colonies were counted after 18 days. The mean number of spheres formed per 96-well plate is shown +/- standard deviation (SD), as well as the mean number of colony forming units (CFUs) +/- SD per cm2 from a triplicate determination. (6.16 MB TIF) [file pone.0013474.s002.tif]

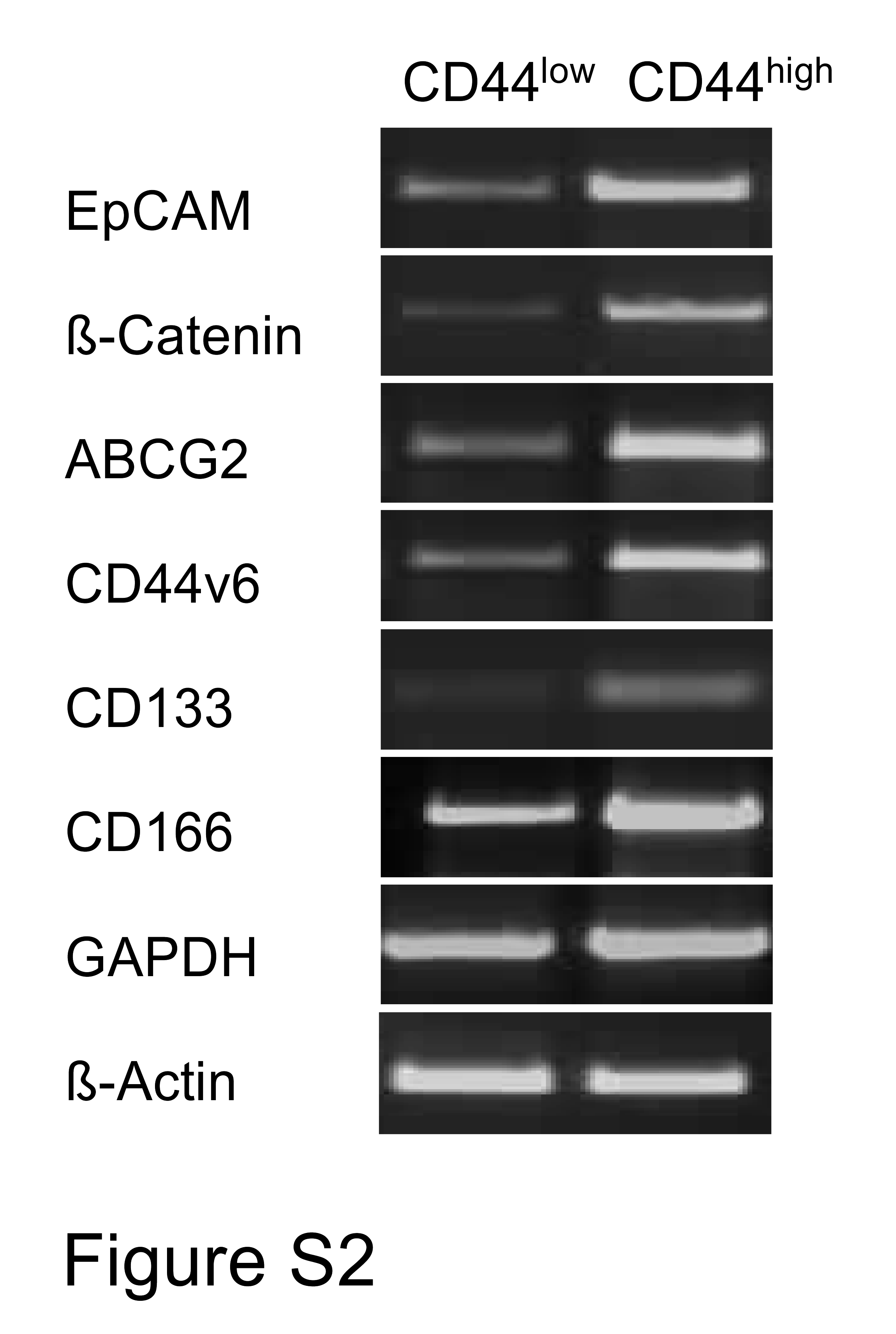

Supplement: Figure S2 — Enhanced expression of cancer stem cell markers in colorectal HT-29 CD44high/CD24high/EpCAMhigh cells. CD44high/CD24high/EpCAMhigh and CD44low/CD24low/EpCAMlow HT-29 cancer cells isolated from mouse xenograft have been analyzed for EpCAM, Β-catenin, ABCG2, CD44v6, CD133 and CD166 by semiquantitative RT-PCR. (0.54 MB TIF) [file pone.0013474.s003.tif]

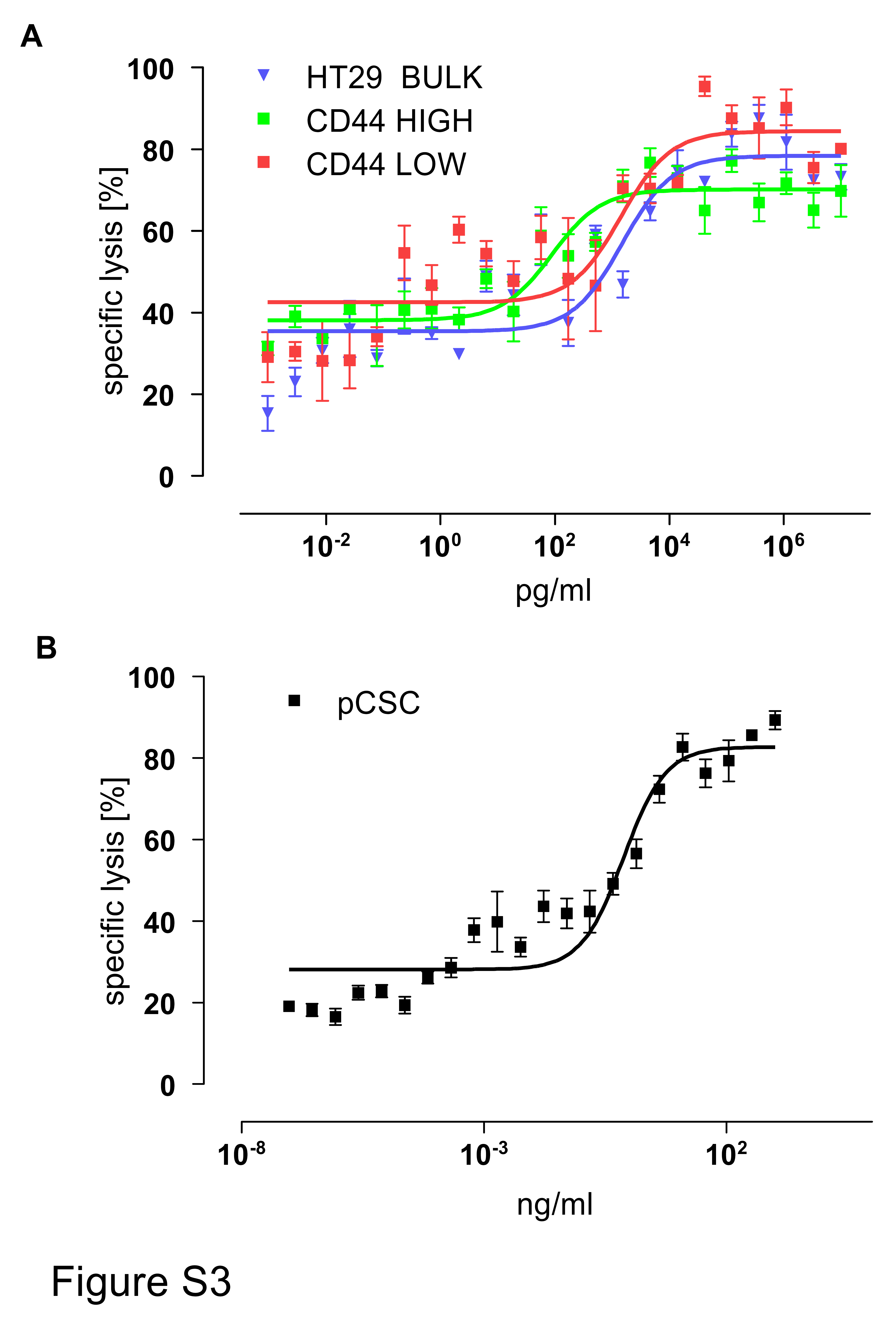

Supplement: Figure S3 — Cytotoxicity assays using HT-29 xenograft-derived and primary tumor-initiating target cells. (A) HT-29 xenograft-derived bulk cells and cells sorted for CD44high/CD24high/EpCAMhigh and CD44low/CD24low/EpCAMlow were analyzed by 51Cr-release standard cytotoxicity assay. (B) TICs were analyzed by 51Cr-release standard cytotoxicity assay. CD8-enriched T cells were used at an E:T ratio of 10:1. Results from triplicate determinations are shown as mean values with standard deviations. (1.65 MB TIF) [file pone.0013474.s004.tif]

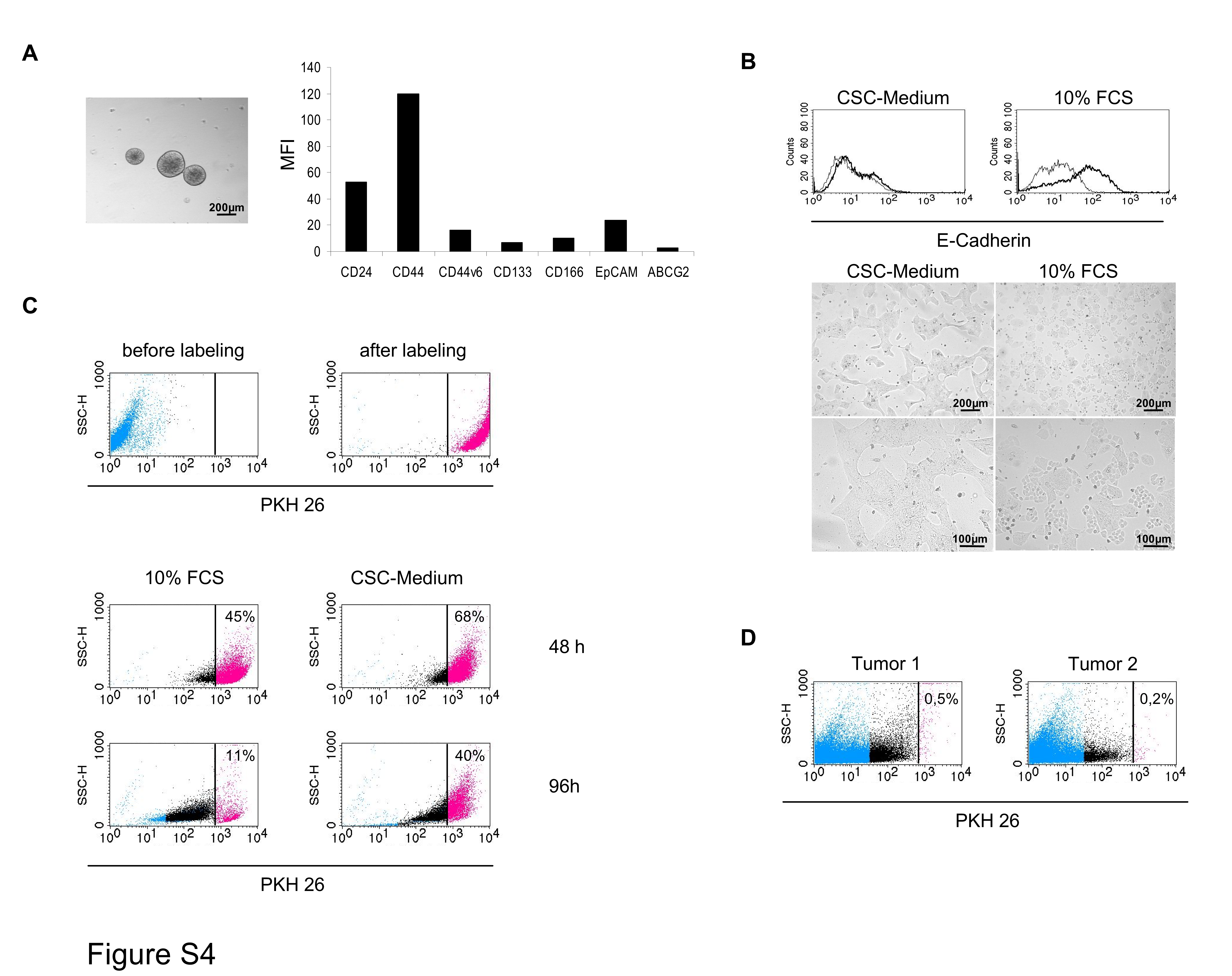

Supplement: Figure S4 — Characterization of TICs for their cancer stem cell phenotype. (A) Sphere formation (left panel) and FACS analysis of cancer stem cells for markers by FACS analysis. MFI-values relative to isotype control are shown (right panel). (B) TICs were cultured under serum-free conditions in TIC medium or under differentiation conditions in RPMI supplemented with 10% FCS in collagen-coated culture flasks. Upper panel: FACS staining of E-cadherin (black line) vs. control (grey line) and lower panel: microphotographs of TICs cultured under FCS and TIC growth conditions taken at 40x and 100x magnification. (C) TICs cultured under serum-free conditions (TIC medium) and differentiation conditions (RPMI supplemented with 10% FCS) analyzed for PKH-26 staining by FACS after 48 and 96 h. (D) PKH-26 labeled TICs were engrafted into NOD/SCID mice. After 4 weeks, the retention of PKH-26 label was determined on TICs by FACS after tumor digestion. (9.70 MB TIF) [file pone.0013474.s005.tif]

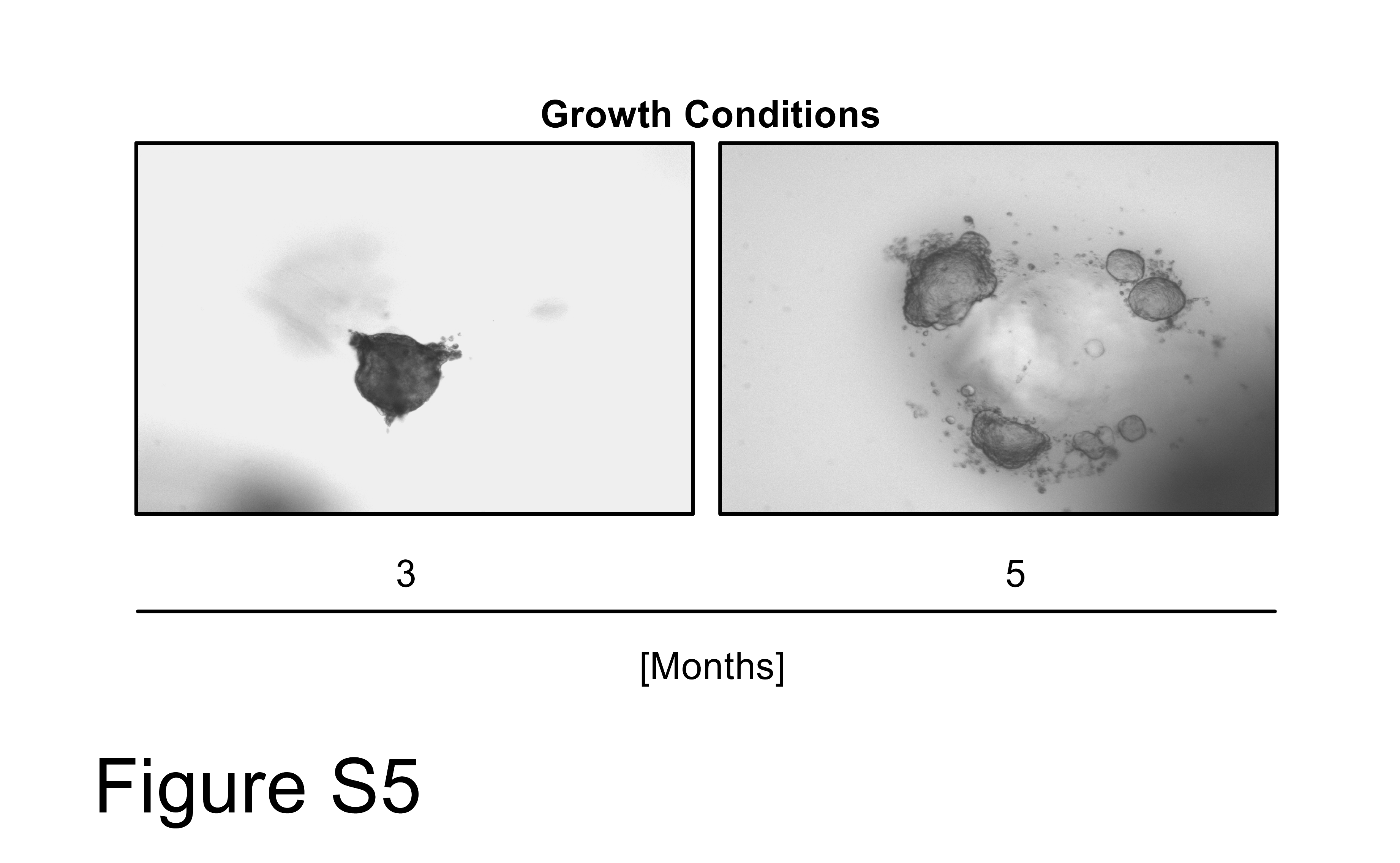

Supplement: Figure S5 — Soft agar still supports colony growth after a long time. Colony growth by TICs inoculated into 3- or 5-month old soft agar. (2.11 MB TIF) [file pone.0013474.s006.tif]
